# Patient Engagement in Health Management as a Mediator Between Perceived Risk and COVID-19 Related Distress in Patients With IBD: A Structural Equation Model

**DOI:** 10.3389/fpsyt.2021.733544

**Published:** 2021-10-27

**Authors:** Greta Castellini, Lorenzo Palamenghi, Mariarosaria Savarese, Serena Barello, Salvatore Leone, Enrica Previtali, Alessandro Armuzzi, Guendalina Graffigna

**Affiliations:** ^1^EngageMinds HUB–Consumer, Food & Health Engagement Research Center, Università Cattolica del Sacro Cuore, Milan, Italy; ^2^Faculty of Agriculture, Food and Environmental Sciences, Università Cattolica del Sacro Cuore, Cremona, Italy; ^3^Department of Psychology, Università Cattolica del Sacro Cuore, Milan, Italy; ^4^Faculty of Psychology, Università Cattolica del Sacro Cuore, Milan, Italy; ^5^AMICI Onlus, Associazione Nazionale per le Malattie Infiammatorie Croniche dell'Intestino, Milan, Italy; ^6^Fondazione Policlinico Universitario A. Gemelli IRCCS, Roma, Italy

**Keywords:** COVID-19, inflammatory bowel disease, patient engagement, stress, structural equation model-SEM

## Abstract

**Objective:** This study aimed to evaluate the impact of the COVID-19 emergency on patients with IBD's psychological distress, understanding the role of patient engagement as a mediator.

**Methods:** An online questionnaire was created, measuring perceived risk susceptibility toward COVID-19, perceived stress, and patient engagement. The questionnaire was distributed to a purposive sample of IBD patients who belonged to the Italian Association for patients with IBD (AMICI Onlus) in April 2020. Structural equation models were implemented.

**Results:** The effect of the perceived risk susceptibility toward COVID-19 contagion on the perceived stress is fully mediated by patient engagement (β = 0.306, *p* < 0.001). Moreover, the patient engagement mitigates the perceived stress (β = −0.748, *p* < 0.001) in our sample of IBD patients, and it is negatively influenced by the perceived risk susceptibility toward COVID-19 (β = −0.410, *p* < 0.001).

**Conclusion:** Patient engagement is the key factor that explains how the perceived risk susceptibility toward COVID-19 affects the perceived psychological distress in patients with IBD, underlining that the perceived risk of contagion increases their perceived level of stress through a decrease of patient engagement.

## Introduction

On February 21, 2020, the pandemic of COVID-19 started to spread in Italy, with the first group of cases identified in the northern part of the country. In a few weeks, the disease spread all over the country: by the end of March, there were already more the 100,000 total cases, with more than 11,000 deaths. The pandemic itself, the constant media reports of deaths and new cases, and the measures enacted to slow down the spread (e.g., lockdown, wearing gloves and masks, social distancing, etc.) exerted an important psychological impact on the general population (1), also in terms of anxiety, depression, and distress (2–4).

These psychological outcomes are particularly relevant for more fragile populations, such as chronic patients: for instance, it is known that patients with inflammatory bowel diseases (IBD) normally have higher levels of general psychological distress when compared to the general population (5), in particular depending on the disease activity (6). Additionally, as previous research shows, patients with IBD—and in particular those with comorbidities or a suppressed immune system—have a high fear of infection and reported leaving their homes less (e.g., for going at the supermarket) (7), which combined with preventive measures enacted to prevent the spread of COVID-19 such as quarantines and lockdown, may have forced a change in daily habits of patients with IBD, potentially impacting their psychological ability to engage in effective health management.

This combination of high-perceived risk of infection and change in daily patterns may have exerted an important impact on patients with IBD's psychological well-being and, in particular, on their levels of perceived psychological distress. Perceived psychological distress should be monitored with caution in patients with IBD, as it is known to be associated with an increased disease activity (8).

It is then important to evaluate the impact that the COVID-19 emergency and, in particular, the perceived risk of infection may have exerted on patients with IBD's psychological distress, as understanding the dynamic behind this stressor-outcome relationship will help tailor more effective and targeted interventions to ease the burden. For this very reason, it is also important to understand whether there are other subjective, psychological characteristics of the patients that can help explain the existing relationship between patients' perceived risk of infection and the perceived psychological distress. Our claim is that the patients' engagement in health management during this COVID-19 emergency mediates the relationship between the perceived susceptibility to COVID-19 and the generated perceived distress. The ability to deal with a difficult situation and the consequent psychological adjustment, in fact, have been demonstrated to be mediators between risk perception and psychological well-being or other psychological outcomes (9, 10).

This could be due to the fact that a higher degree of perceived health risk (namely, in the case of COVID-19, the perceived susceptibility to the infection) could disrupt the psychological readiness of the patients to engage in one's health management and adapt to the new situation. In turn, it is well known that perception to be unable to actively engage in one's health management in unexpected situations—together with the consequent negative emotions elicited from this experience—can lead to high levels of psychological distress (11, 12). In particular, the research carried out by Liu et al. (13) during the COVID-19 emergency demonstrated that the psychological adaptation could be an important factor in identifying stress-prone individuals during a pandemic. Conversely, a more engaged patient has been identified as less distressed in response to a perceived risk or sudden change (14) and more capable of managing his own health (15), which are aspects particularly challenged by this pandemic. In particular, previous studies revealed that a higher level of patient engagement promotes important outcomes for IBD patients, such as an increased health-related quality of life (16), and has been proposed as an important ingredient to promote IBD patients' psychosocial wellbeing (17). Finally, a more engaged patient has been found to better cope with negative emotions and panic, even during quarantine (18, 19).

**Figure 1:**
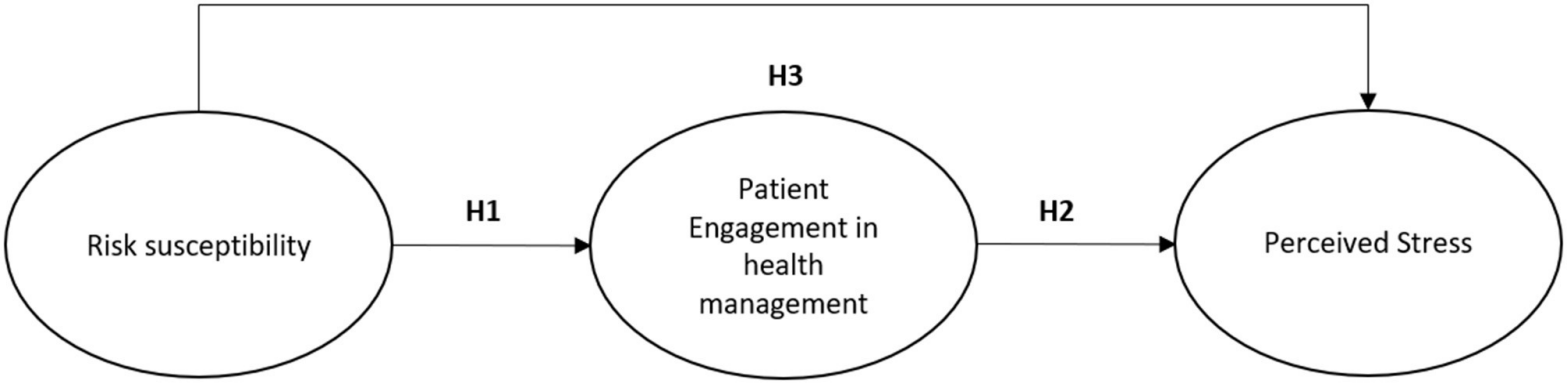
The hypothesized model.

However, little is known about this aspect in patients with IBD in the specific context of COVID-19 emergency. Following from these premises, our hypotheses are that (Figure 1):

1. A higher level of perceived risk susceptibility toward COVID-19 contagion corresponds to a lower level of patient engagement in one's own health management during the COVID-19 emergency;
2. A higher level of patient engagement in one's own health management during the COVID-19 emergency is a precursor of a lower level of perceived stress;
3. As a consequence of the previous hypotheses, the study assumes that the effect of the perceived risk susceptibility toward COVID-19 contagion on the perceived stress is mediated, at least partially, by the patient engagement in one's own health management.

## Materials and Methods

### Procedure and Sample

This is a cross-sectional study that used a CAWI (Computer Assisted Web Interviewing) methodology. Data were collected using a questionnaire distributed between April 6 and April 13, 2020, to a mailing list of a purposive sample of patients who belonged to the Italian Association for Patients with IBD–AMICI Onlus. The questionnaire was sent to 4,187 patients with IBD subscribed to AMICI Onlus mailing list who were over 18 years old. A total of 1,014 (response rate 24%) were returned completed and they were used for the statistical analyses. All participants provided informed consent at the beginning of the questionnaire. The study was approved by the Ethical Commission of the Catholic University of the Sacred Heart (CERPS) and was conducted in accordance to the ethical standards of the Declaration of Helsinki and its following amendments.

### Measures

In order to answer the research questions, the survey involved the measurement of the following variables:

- *Socio-demographic variables:* a series of socio-demographical data were collected, including age, gender, education, urban center size, geographical area, and marital status in order to describe the sample.
- *Disease characteristics of the patients:* some data relating to the year of diagnosis and the type of disease (Crohn's disease, Ulcerative colitis, IBD unclassified) were collected.
- *Perceived risk susceptibility toward COVID-19 contagion:* participants were asked to rate from 1 (*very little*) to 5 (*a lot*) their perceived risk of being personally infected by the new COVID-19 virus. Since the question was considered particularly sensitive, participants were also granted the possibility to answer “I'd rather not answer.”
- *Perceived stress*: this variable was measured using the Perceived Stress Scale (PSS) that was used in its 4-items version, validated by Cohen and colleagues (20). The PSS is designed to measure the experienced levels of stress caused by a stressful situation. All items were assessed on a 5-point Likert scale ranging from 0 (*never*) to 4 (*very often*). The higher score on this scale represents greater experienced levels of stress caused by a stressful situation. An example of item is, “In the last month, how often have you felt that you were unable to control the important things in your life?”
- *The engagement in one's own health management during the COVID-19 emergency*: This aspect was measures using the Patient Health Engagement Scale (PHE-s®), a measure that, developed according to the Patient Health Engagement model (21), assesses people health engagement level, defined as the “*people psychological readiness and sense of mastery to become active player in their own health management and health risk prevention*.” Previous studies demonstrated its robust psychometric properties (1). This scale is composed by 5 items measured on a 7-point Likert scale. Those who have higher score on this scale are completely aware of the characteristics and consequences of their disease condition, and assume a more responsible position in their behaviors and disease management experienced. In this research a slightly adapted version of the scale to the specific COVID-19 situation was adopted (22). The questionnaire's items are available in the Supplementary Materials (Online Resource 1).

### Data Analysis

Descriptive statistics were computed for each item (symmetry, kurtosis, mean, median, and standard deviation), and normality of distribution was checked. As suggested by Anderson and Gerbing (23) in order to check the adequacy of the measurement model a confirmatory factor analysis (CFA) was run using MPLUS 8. The models were estimated using Satorra-Bentler Correction (MLM) and evaluated using the chi-square (i.e., non-significant values of *p*-value indicate a good model) and approximate fit statistics, based on Hu and Bentler (24). These included Root Mean Square Error of Approximation (RMSEA) <0.08; Confirmatory Fit Index (CFI) ≥0.90; and Tucker-Lewis Index (TLI) ≥0.90. Moreover, structural equation modeling (SEM) was used to analyze the relationships between the perceived risk susceptibility toward COVID-19 contagion and the patient engagement in one's own health management during the COVID-19 emergency on perceived stress. In particular, the bootstrap technique (25) was used in order to confirm the mediation hypothesis (the indirect relationship between an independent variable and the dependent variable considering the presence of the mediator) with more statistical rigor than the Sobel test (26, 27). The Percentile bootstrapping was performed at a 95% confidence interval with 5,000 bootstrap samples (28).

Finally, a power analysis was conducted to understand whether the study sample size was adequate for the planned analyses. Since the sample size required for SEM depends on multiple factors not considered in rule-of-thumbs guidelines [i.e., the number of latent factors, the number of indicators, and the magnitude of factor loadings and correlations; (29)], we decided to use the pwrSEM app on Shiny (30) based on Monte Carlo simulation with 1,000 repetitions to estimate the power for the regression paths in our hypothesized model. The factor loadings of PSS and of PHE-s® were, respectively, set at 0.63 and at 0.69, and they were estimated using the Spearman-Brown prophecy formula that considers the scales' overall reliability to estimate the average factor loading strength of individual items in that measure. The results revealed that, with the sample size of 1,014 and alpha level of 0.05, the test has at least 93% power to detect indirect effect sizes equal to or larger than 0.03.

## Results

### Sample Characteristics

The sample is made up of 1,014 Italian patients with IBD of which 476 (46.9%) are male and 538 (53.1%) are female with an age between 18 and 84 years with an average of 48.35 years and a standard deviation of ±13.20. The demographic profile is presented in detail in Table 1.

**Table 1:** Demographic and disease characteristics of the sample (*N* = 1,014).

	***n***	**%**
**Gender**		
Male	476	46.9
Female	538	53.1
**Age**		
18–25	52	5.1
26–35	131	12.9
36–45	225	22.2
46–55	279	27.5
56–65	232	22.9
> 66	95	9.4
**Education**		
No qualifications	2	0.2
Elementary	5	0.5
Junior high	141	13.9
Senior high	513	50.6
College or university	301	29.7
Master/PhD	52	5.1
**Urban center size**		
Up to 5,000 inhabitants	158	15.6
5/10,000 inhabitants	149	14.7
10/30,000 inhabitants	217	21.4
30/100,000 inhabitants	173	17.1
100/500,000 inhabitants	135	13.3
More than 500,000 inhabitants	121	11.9
Missing	61	6.0
**Geographic area**		
North-West	295	29.1
North-East	360	35.1
Center	163	16.1
South and Islands	196	19.8
**Marital status**		
Unmarried	268	26.4
Married/cohabitant	660	65.1
Divorced	72	7.1
Widower/widow	14	1.4
**Net monthly income**		
Up to 600 euro	17	1.7
601–900 euro	27	2.7
901–1,200 euro	57	5.6
1,201–1,500 euro	118	11.6
1,501–1,800 euro	96	9.5
1,801–2,500 euro	177	17.5
2,501–3,500 euro	190	18.7
3,501–4,500 euro	59	5.8
More than 4,500 euro	37	3.6
Missing	236	23.3
**Chronic bowel disease**		
Crohn's disease	508	50.1
Ulcerative colitis	490	48.3
Unclassified IBD	16	1.6
**Year of diagnosis**		
1970–1979	18	1.8
1980–1989	108	10.7
1990–1999	212	20.9
2000–2009	312	30.8
2010–2020	364	35.9

### The Measurement Model

Means, standard deviations, medians, symmetry, and kurtosis of all the scales and items used in the model were calculated, showing that all the items were normally distributed, with the only exception of the last item of the Scale (PHE-s®) COVID-19 version (PHE_Item 5) (Table 2).

**Table 2:** Descriptive statistics of items.

**Item**	**M**	**SD**	**Md**	**A**	**K**
**Perceived stress scale (PSS)**					
PSS_ Item 1	1.68	0.98	2.00	0.06	-0.37
PSS_ Item 2	1.34	0.82	1.00	0.64	0.85
PSS_ Item 3	1.87	0.84	2.00	0.24	0.21
PSS_ Item 4	1.46	0.99	1.00	0.32	-0.41
**Scale (PHE-s** ^®^ **) COVID-19 version**					
PHE_ Item 1	4.50	1.56	5.00	−0.16	-0.47
PHE_ Item 2	4.93	1.42	5.00	−0.23	-0.35
PHE_ Item 3	5.26	1.75	5.00	−0.63	-0.62
PHE_ Item 4	4.72	1.67	5.00	−0.20	-0.76
PHE_ Item 5	5.41	1.12	5.00	−0.52	1.84
**Risk susceptibility**					
In particular, how much do you feel at risk of being infected by the new Coronavirus?	3.66	0.90	1.00	−0.34	-0.29

*N = 1,014; M, mean; SD, standard deviation; Md, median; A, asymmetry; K, kurtosis*.

Moreover, a confirmatory factor analysis (CFA) was carried out to understand whether the data conformed to the assumption that these latent variables (perceived stress and the patient engagement in one's own health management during the COVID-19 emergency) represent two separated constructs, validating the measurement model. For this purpose, the MLM method was used.

To determine goodness of fit, Beavers (31) proposed that the factor loadings <0.40 are weak and factor loadings >0.60 can be considered strong. Moreover, the acceptable threshold value for composite reliability (CR) is above 0.70, while that for average variance extracted (AVE) is above 0.50 (32). Results from the CFA confirmed the hypothesized two factor measurement model and also all of the loadings of the observed variables on the latent variables were also significant, revealing that all of the latent constructs were well operationalized by their indicators even if the AVE relating the Perceived Stress Scale is slightly under below the threshold (Table 3).

**Table 3:** Confirmatory factor analysis properties.

	**Stand. Factor loadings**	**SE**	***P***	**α**	**CR**	**AVE**
Perceived stress scale (PSS):				0.76	0.76	0.44
PSS_ Item 1	0.63	0.03	^***^			
PSS_ Item 2	0.66	0.03	^***^			
PSS_ Item 3	0.66	0.02	^***^			
PSS_ Item 4	0.71	0.02	^***^			
Scale (PHE-s®) COVID-19 version				0.83	0.84	0.54
PHE_ Item 1	0.75	0.02	^***^			
PHE_ Item 2	0.76	0.02	^***^			
PHE_ Item 3	0.64	0.02	^***^			
PHE_ Item 4	0.77	0.02	^***^			
PHE_ Item 5	0.69	0.02	^***^			

*** *p < 0.001; N = 1,014; χ^2^ = 165.904; df = 26; p < 0.001; CFI = 0.96; TLI = 0.94; RMSEA = 0.073 (LO90 = 0.062, HI90 = 0.084). CR, composite reliability; AVE, average variance extracted; α = Cronbach alpha*.

### The Structural Model

**Figure 2:**
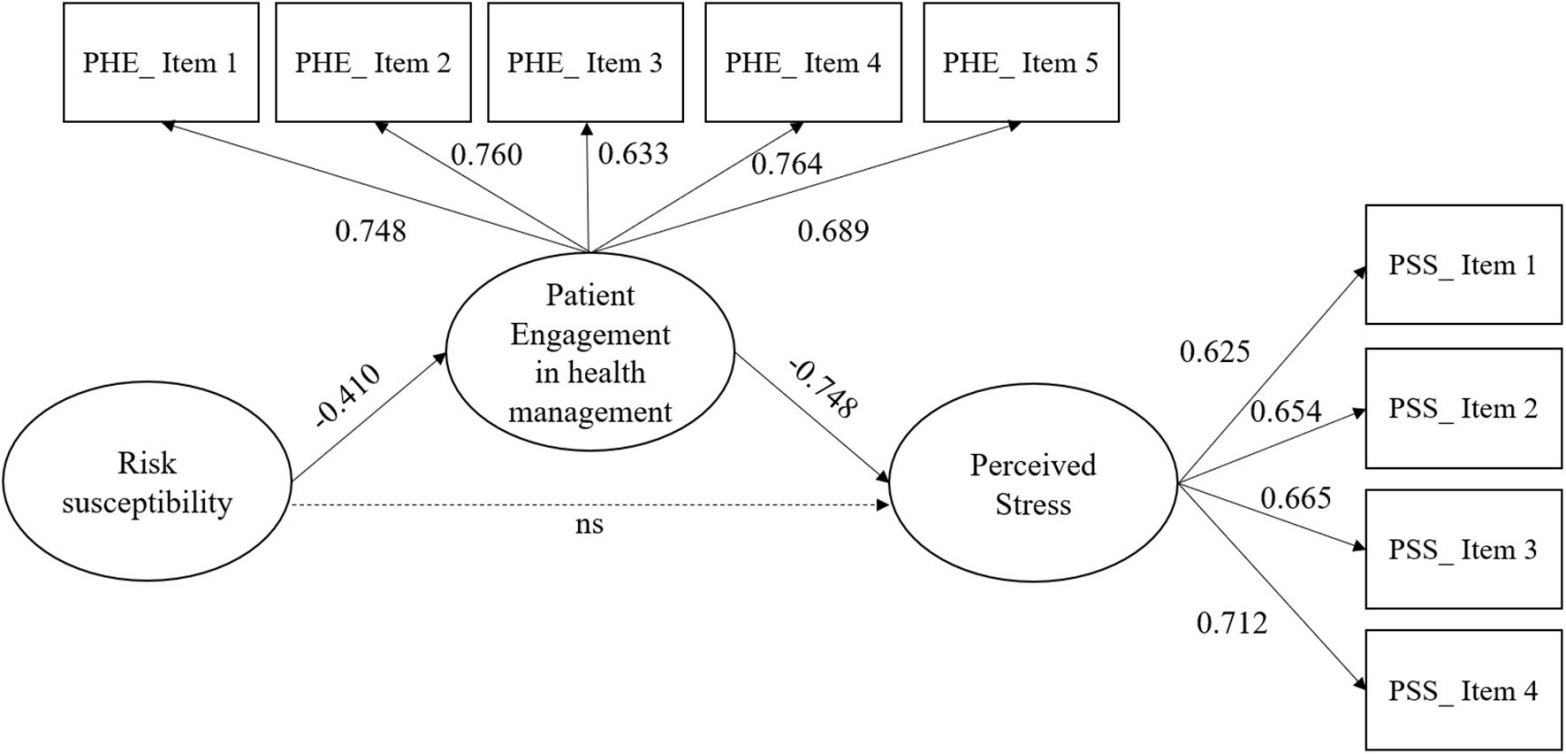
The results of structural equation model (SEM). ns, not significant.

Finally, a structural equation model (SEM) was run in MPLUS 8 to assess the presented model. The model provided a very good fit to the data: χ^2^ = 170.102; df = 33; *p* ≤ 0.001; CFI = 0.96; TLI = 0.94; RMSEA = 0.065 (LO90 = 0.055, HI90 = 0.074). In accordance with the hypothesis, the patient engagement in one's own health management during the COVID-19 emergency was negatively influenced by the perceived risk susceptibility toward COVID-19 contagion (β = −0.410, *p* < 0.001), confirming hypothesis 1, and the perceived stress was negatively influenced by the patient engagement in one's own health management during the COVID-19 emergency (β = −0.748, *p* < 0.001), confirming hypothesis 2 (see Figure 2).

In Table 4, the results of the total effect, indirect effect, and direct effect are shown. We note that the patient engagement in one's own health management during the COVID-19 emergency plays a full mediating role in the relationships between the perceived risk susceptibility toward COVID-19 contagion and perceived stress (indirect effect = 0.306, CI = 0.254; 0.361). Hence, hypothesis 3 is supported by the results.

**Table 4:** Standardized indirect effect of the model.

			**Bootstrapping**	
			**Percentile Bootstrapping 95% CI (confidence interval) of the coefficients**	
	**Point estimate**	**SE**	**Lower**	**Upper**
**Total effect**				
Risk susceptibility → perceived stress	0.28	0.04	0.20	0.35
**Indirect effect**				
Risk susceptibility → patient engagement in health management → stress	0.31	0.03	0.25	0.36
**Direct effect**				
Risk susceptibility → perceived stress	−0.03	0.04	−0.10	0.04

*N = 998; SE, Standard Error; Mediator: interpersonal influence; Estimating of 5,000 bootstrap sample*.

## Discussion

The results from the confirmatory factor analyses show that the measures used to assess the patient engagement in one's own health management during the COVID-19 emergency and perceived stress were reliable.

The analyses conducted via the structural equation model, indeed, confirmed our initial hypotheses. In particular, our research showed that higher levels of perceived risk of contagion from COVID-19 negatively affects the ability of patients with IBD to psychologically engage in their health management. Recent research carried out on the Italian population (33) of patients with IBD confirmed these results, showing that patients with IBD with higher levels of perceived risk of contagion from COVID-19 have significantly lower levels of patient engagement. In line with these results, other research on chronic diseases demonstrated that the impact of the perception of risk on patient engagement could lead to inadequate preventive behaviors (14, 34, 35). Our results also suggest that in the case of Italian patients with IBD coping with their disease during COVID-19 emergency, the perception of risk of contagion impacts the ability to engage in their health management. Investigating this relation is indeed necessary to support patients in dealing with the perception of risk contagion and prevent possible misconducts, such as under- or over-estimation of the importance of preventive measures.

Moreover, this research demonstrated that in the Italian patients with IBD the ability to engage in health management during this emergency influences the level of perceived stress. These findings are supported by other studies that pointed out that the level of stress and anxiety experienced by patients are significantly correlated with the level of engagement that patients have of their disease (36, 37). Our results also showed that in patients with IBD the perceived susceptibility to COVID-19 has a positive relationship with perceived psychological distress, hence showing that those patients who feel more vulnerable to COVID-19 also show higher levels of psychological distress. These results have also been confirmed by other studies that have tried to understand the connection between the risk of contagion from COVID-19 and perceived stress (38, 39), on different patients. In Italy, a research carried out by Di Crosta et al. (40) on the healthy Italian population showed that higher levels of risk of contagion by COVID-19 could significantly impact on the level of perceived stress and its symptoms (40). These results suggest to monitor patients' susceptibility to stressful events as they may manifest worse symptoms and experiment poorer quality of life.

Finally, our results highlighted the key role of Patient Health Engagement, which fully mediates the relationship between perceived risk of contagion from COVID-19 and level of perceived stress in patients with IBD. These results underlined that the perceived risk impacts on the ability of patients with IBD to be psychologically engaged in their health management which, in turn, affects the level of perceived stress. In other words, this means that the risk of contagion from COVID-19 increases the level of stress through the decrease of the patient engagement in health management. These results are important from both a scientific and a pragmatic point of view. From a scientific point of view, they explained the relationship between level of perceived stress and risk of contagion from COVID-19 in patients with IBD. In particular, this study highlighted the key role of the patient engagement in influencing the level of stress, showing how risk of contagion from COVID-19 has only an indirect effect on it. However, most studies have investigated the direct relationship between risk of contagion from COVID-19 and perceived stress without deepening the process that links these two psychological phenomena (41, 42). Moreover, these findings have some practical implications. Specifically, they showed how important it is for healthcare professionals, involved in IBD patient care, to monitor patients' perceived risk of contagion from COVID-19 in order to avoid worsening in their health management ability which, in turn, can cause high levels of stress. Indeed, high level of stress in patients with IBD can determine negative health outcomes (8, 43) and a poorer health-related quality of life (44). Furthermore, it is important not only to monitor but try to contain the risk of contagion from COVID-19 perceived by patients with IBD. In particular, some studies (45, 46) showed how through a clear and effective communication that describes the preventive actions necessary to reduce the infection from COVID-19 it is possible to reduce the perception of risk felt by people and the consequent negative effects.

Nonetheless, the present study has several limitations: first, the level of psychological distress (as well as the other variables) was assessed through self-report measures, which may suffer from biases. However, through self-reported measures it is possible to assess patients' direct experience, which is required in a context that calls for giving voice to the patients, for example, through patient-reported measures of experiences and outcomes. For this reason, we have anyway privileged for this type of data collection. In addition, the PSS scale was adapted from English, and although the CFA showed a good reliability, a proper Italian validation should be carried out to assess the translated version's psychometric properties. Moreover, the sample was recruited with a non-random method, which may have hampered its representativeness of the Italian population of patients with IBD. The patients involved in our research, indeed, belonged to the Italian Association for Patients with IBD: on the one hand, it can be a limitation of the study, on the other this sample has a very similar care experience and they can be considered as expert patients. The participation to the survey was free, so the patients who answered were convinced and inclined to complete the questionnaire.

## Conclusion

Our study has allowed an understanding of the process that explains the relationship between the risk of contagion from COVID-19 and the level of stress perceived by patients with IBD during this emergency situation. Understanding this psychological process is of paramount importance for these patients because, as shown by previous studies, high levels of perceived stress could lead to an increase in anxiety and depressive states causing worsening not only of their quality of life but also of their intestinal symptoms. The innovative result of this research is that the perceived risk of contagion from COVID-19 increases the level of stress of patients with IBD through the decrease of their engagement in health management. Consequently, from a scientific point of view, this study allows us to explain the psychological process that links the risk of contagion from COVID-19 to perceived stress in patients with IBD by showing how the patient engagement in health management is the key variable that directly affects perceived stress while the level of perceived risk influences it indirectly. Furthermore, these results highlight the importance of monitoring and managing the risk of contagion from COVID-19 perceived by patients with IBD to avoid increasing stress levels through a decrease in their health management capacity.

## Data Availability Statement

The raw data supporting the conclusions of this article will be made available by the authors, without undue reservation.

## Ethics Statement

The studies involving human participants were reviewed and approved by the study was approved by the Ethical Commission of the Catholic University of the Sacred Heart (CERPS), and was conducted in accordance to the ethical standards of the Declaration of Helsinki and its following amendments. The patients/participants provided their written informed consent to participate in this study.

## Author Contributions

MS, SL, EP, AA, and GG: conceptualization. MS, GC, and GG: methodology. GC: formal analysis and data curation. GC, LP, and SB: writing—original draft preparation. LP, SB, GC, GG, and MS: writing—review and editing. GG: supervision and project administration. All authors have read and agreed to the published version of the manuscript.

## Conflict of Interest

The authors declare that the research was conducted in the absence of any commercial or financial relationships that could be construed as a potential conflict of interest.

## Publisher's Note

All claims expressed in this article are solely those of the authors and do not necessarily represent those of their affiliated organizations, or those of the publisher, the editors and the reviewers. Any product that may be evaluated in this article, or claim that may be made by its manufacturer, is not guaranteed or endorsed by the publisher.
