# Supplementary material for: Patient Engagement in Health Management as a Mediator Between Perceived Risk and COVID-19 Related Distress in Patients With IBD: A Structural Equation Model
Source: Front Psychiatry. 2021 Oct 27;12:733544. doi: 10.3389/fpsyt.2021.733544 (PMC8578824; doi:10.3389/fpsyt.2021.733544)
Supplement: Supplementary file 1 [file Data_Sheet_1.docx]

Supplementary Material

1. Survey guide

**Gender**

- - Male
  - Female

**Year of birth (in numbers)**

|  |
| --- |

**Region of residence**

- - North-West
  - North-East
  - Center
  - South and Islands

**Inhabited centre size**

- - Up to 5.000 inhabitants
  - 5/10.000 inhabitants
  - 10/30.000 inhabitants
  - 30/100.000 inhabitants
  - 100/500.000 inhabitants
  - More than 500.000 inhabitants
  - I don’t know

**Level of education**

- No qualifications
- Elementary
- Junior high
- Senior high
- College or university
- Master/PhD

**Marital status**

- Unmarried
- Married/cohabitant
- Divorced
- Widower/widow

**Net monthly income**

- - Up to 600 euro
  - 601-900 euro
  - 901-1200 euro
  - 1201-1500 euro
  - 1501-1800 euro
  - 1801-2500 euro
  - 2501-3500 euro
  - 3501-4500 euro
  - More than 4500 euro
  - I prefer not to answer

***Type of disease***

- Crohn's disease
- Ulcerative colitis
- IBD unclassified

***Year of diagnosis _____________***

***How much do you feel at risk of being infected by the new Coronavirus? (1= Not at all 5 = A lot at risk).***

| ***Not at all*** | ***A little*** | ***Nor little nor much*** | ***Quite at risk*** | ***A lot at risk*** |  | ***I don’t have an opinion*** |
| --- | --- | --- | --- | --- | --- | --- |
| ***1*** | ***2*** | ***3*** | ***4*** | ***5*** |  | ***6*** |

***Following, you’ll find 5 statements that describe how a person can feel when thinking about the risk of being infected from the new Coronavirus (COVID-19). Each sentence can be completed by choosing one of the 4 specific states, or the intermediate points between the different states. Please, indicate the position that best indicates your state, by clicking on the corresponding dot.***

***Please, check that you have answered all the statements and that you have indicated only one option for each of them.***

*.*

| *Thinking about the management of your illness in this emergency...* | | | | | | | |  |
| --- | --- | --- | --- | --- | --- | --- | --- | --- |
|  |  |  |  |  |  |  |  |  |
| *1* | I feel like I’m in blackout  O | O | I feel in alert  O | O | I feel informed  O | O | I feel positive  O |  |
| *2* | I feel lost  O | O | I feel alarmed  O | O | I feel conscious  O | O | I feel in peace  O |  |
| *3* | I feel overwhelmed by emotions  O | O | I feel in anxiety every time I hear talking about the Covid-19  O | O | I feel I got used to this emergency  O | O | My life goes on regardless of this situation  O |  |
| *4* | I'm living moments of great discouragement  O | O | I often feel anxious  O | O | I feel I got used to this situation  O | O | I am generally optimist about my future and my health  O |  |
| *5* | I feel paniked  O | O | I feel the urgency to do something  O | O | I keep calm  O | O | I am in control  O |  |

***The following questions are about your emotions and thoughts in the last month. For each question, answer indicating how often you feel or think in a certain way (from 0=never to 4=very often)***

|  | **0 = Never** | **1 = Almost never** | **2 = Sometimes** | **3 = Fairly Often** | **4 = Very Often** |
| --- | --- | --- | --- | --- | --- |
| In the last month, how often have you felt that you were unable to control the important things in your life? | O | O | O | O | O |
| In the last month, how often have you felt confident about your ability to handle your personal problems? | O | O | O | O | O |
| In the last month, how often have you felt that things were going your way? | O | O | O | O | O |
| In the last month, how often have you felt difficulties were piling up so high that you could not overcome them? | O | O | O | O | O |
